# Constructing a prediction model of inflammatory bowel disease recurrence based on factors affecting the quality of life

**DOI:** 10.3389/fmed.2023.1041505

**Published:** 2023-03-09

**Authors:** Maojuan Li, Yan Tao, Yang Sun, Jing Wu, Fengrui Zhang, Yunling Wen, Min Gong, Jingxian Yan, Hao Liang, Xinyu Bai, Junkun Niu, Yinglei Miao

**Affiliations:** ^1^Department of Gastroenterology, The First Affiliated Hospital of Kunming Medical University, Kunming, Yunnan, China; ^2^Yunnan Province Clinical Research Center for Digestive Diseases, Kunming, Yunnan, China

**Keywords:** inflammatory bowel disease, quality of life, influencing factors, recurrence, prediction model

## Abstract

**Aim:**

This study aimed to determine the factors affecting the quality of life of patients with inflammatory bowel disease (IBD) and to construct a disease recurrence prediction model based on these influencing factors.

**Methods:**

A prospective, single-center study in China was conducted between October 2020 and March 2021. The quality of life of patients was assessed using the Inflammatory Bowel Disease Questionnaire (IBDQ). Multiple stepwise regression analysis was used to analyze the factors influencing the quality of life of patients with IBD. The chi-square test and the point-biserial correlation analysis were performed to identify factors associated with clinical recurrence. A binary logistic regression model was constructed to predict the recurrence. The receiver operating characteristic curve was used to evaluate the prediction model. Patients with IBD from April 2021 to June 2021 were randomly included for model verification to evaluate the disease recurrence prediction model.

**Results:**

The average IBDQ score of patients with IBD was 172.2 ± 35.0 (decreased by 23.2%). The scores of all dimensions of the IBDQ were decreased, especially emotional function and systemic symptoms. Disease activity, age, extraintestinal manifestations (EIMs), and annual household income were important factors influencing the IBDQ scores of patients with ulcerative colitis, and these accounted for ~57.0% of the factors affecting the quality of life. Disease activity, EIMs, and occupational stress were important factors influencing the IBDQ scores of patients with Crohn's disease, and they accounted for approximately 75.1% of the factors affecting the quality of life. Annual household income, occupational stress, and IBDQ scores were independent risk factors for recurrence. The area under the curve of the recurrence prediction model was 81.1%. The sensitivity and specificity were 81.7 and 71.7%, respectively. The Youden index of the model was 0.534. The established recurrence prediction model has good discriminant validity in the validation cohort.

**Conclusion:**

The quality of life of patients with IBD was generally poor. The use of factors affecting the quality of life to predict disease recurrence has high predictive value and can support the management of IBD by selecting patients at a higher risk for relapse.

## Introduction

Inflammatory bowel disease (IBD) is a chronic inflammatory disorder of the gastrointestinal tract that is believed to be caused by an inappropriate inflammatory response to intestinal microbes in a genetically susceptible host. The most common forms of IBD include ulcerative colitis (UC) and Crohn's disease (CD) (1). IBD is a global disease, and its incidence and prevalence are still increasing worldwide (2, 3). The incidence of IBD in China is generally lower than that in developed countries, such as Western Europe and North America, but epidemiological studies show a significant increase in the incidence of IBD in China (4). The most common clinical symptoms of IBD are abdominal pain, diarrhea, and bloody stools, often accompanied by extraintestinal manifestations (EIMs). Some patients also suffer from complications such as abscesses, fistulas, and stenosis (5).

Due to its chronic, relapsing nature and therapeutic complexity, IBD requires long-term medical management and imposes significant costs on individuals and society. Several studies have reported that patients with IBD have lower employment rates and a higher percentage of work disability than the general population (6). With long-term medical care and productivity loss, the economic burden of patients with IBD is increasing (7). In addition, IBD has been shown to have a psychological impact on daily life, with a significant increase in the incidence of psychological disorders (8). This psychological load adds to the physical burden of the disease and is associated with direct and indirect costs (9). In short, the physical, economic, and psychological burden, as well as the progression of the disease, all affect the quality of life of patients (10, 11). A previous study revealed that one-third of patients with UC relapse within the first year of diagnosis and 70–82% of the patients relapse within 5 years. Additionally, 85% of patients with CD relapse at least one time within 5 years from diagnosis (10). Since patients with severe relapse need intensive treatment, which may accrue high costs and increase the risk of adverse events, detection of relapse and early therapeutic interventions before the severe progression of activity are desirable (12). The current treatment strategies for patients with IBD not only alleviate symptoms and reduce complications but also improve the quality of life and reduce recurrence rates (13).

In the past few years, more attention has been paid to the role of age, C-reactive protein (CRP), fecal calprotectin (FC), erythrocyte sedimentation rate (ESR), endoscopy, EIMs, diet, and pathological scores in the prediction of recurrence (14–17). However, the predictive values of these different parameters in identifying patients at risk of recurrence have been disappointing. Some studies indicated that standard laboratory parameters (e.g., CRP) did not prove to be useful predictors of clinical relapse in IBD as a whole (18, 19). In those studies, the predictive value of FC in patients with UC with clinical remission for relapse was not so prominent (AUC = 0.60–0.70) (20). The predictability of recurrence in CD has also been reported, and the ability to predict recurrence at 1 year was slightly higher than that in UC (AUC = 0.75–0.79) (21, 22). However, due to its relatively high cost, FC is not so frequently measured in patients. In addition, endoscopy for this purpose is sometimes invasive and burdensome for patients (12).

Therefore, prediction tools for disease recurrence need to be developed. The construction of a prediction model of recurrence based on the factors affecting the quality of life of patients with IBD is a new exploration. Based on data from an IBD center in Southwest China, we reported factors influencing the quality of life of patients with IBD and constructed a model to predict disease recurrence, which is of great value for understanding the characteristics of patients with IBD in China and improving their quality of life and outcomes.

## Methods

### Patients

**Figure 1:**
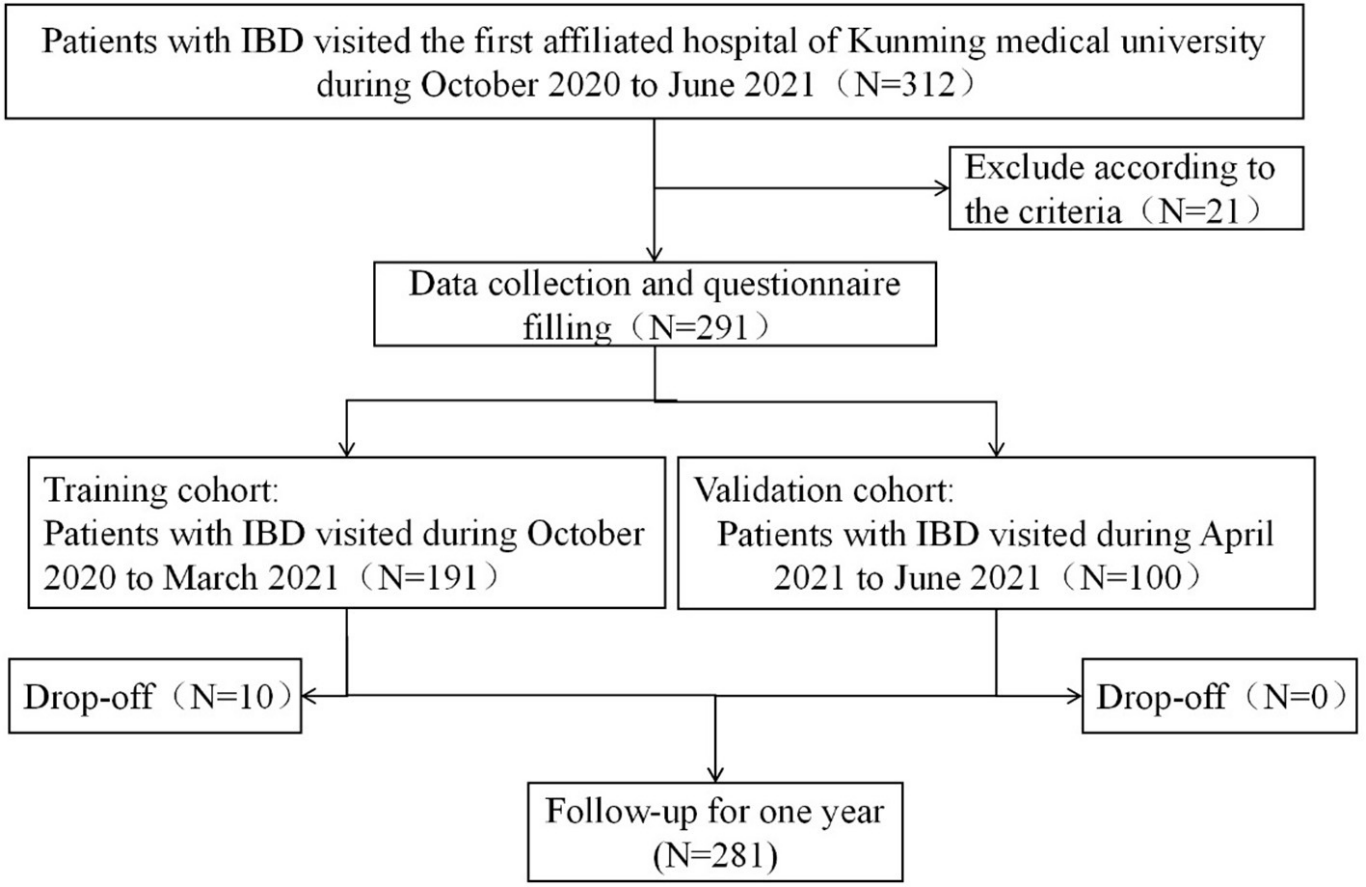
Flowchart summarizing the patients' selection process.

This study was performed in the First Affiliated Hospital of Kunming Medical University, which is a tertiary hospital and an IBD center in Southwest China. We recruited patients with IBD who were hospitalized between October 2020 and March 2021 as a training cohort. Patients with IBD diagnosed from April 2021 to June 2021 were selected to establish a validation cohort. The inclusion criteria were as follows: (1) the diagnosis was confirmed according to the ECCO guidelines (23, 24) and (2) patients gave informed consent. The exclusion criteria were as follows: (1) patients with severe cognitive and mental disorders and (2) patients with other chronic serious diseases, such as heart, kidney, or liver failure, stroke, and serious lung disease. We followed the participants for 1 year and identified recurrence through telephone appointments and outpatient visits. The process for patient inclusion and exclusion is presented in Figure 1.

### Data collection

Demographics, including age, gender, nature of occupation, occupational stress, education level, marital status, family size, annual family income, and type of medical insurance, were obtained by a general information questionnaire. Clinical information, including diagnosis, disease activity, disease localization (the Montreal classification of IBD) (25), disease duration, EIMs (e.g., peripheral and axial arthritis, pyoderma gangrenosum, erythema nodosum, Sweet syndrome, aphthous stomatitis, primary sclerosing cholangitis and episcleritis, anterior uveitis, and iritis), complications (e.g., intestinal obstruction or perforation, massive hemorrhage of the gastrointestinal tract, colon cancer, or toxic megacolon), intestinal surgery, and biologics (e.g., infliximab, vedolizumab, adalimumab, and ustekinumab) were collected from medical records. The disease activity of UC was assessed using the Mayo scores (a Mayo score of ≤ 2 with no single item score of >1 was classified as clinical remission, ranging between 3 and 5 as mild activity, ranging between 6 and 10 as moderate activity, and ranging between 11 and 12 as severe activity) (26). Clinical relapse was defined as the occurrence of symptoms accompanied by an increase in the partial Mayo score of 2 or more, which required a change in therapy, such as the escalation of ongoing therapy with the introduction of steroids and/or immunosuppressive biological drugs (27). The Crohn's disease activity index (CDAI) was used to assess disease activity in CD (CDAI < 150 was classified as remission, 150 to 220 as mild activity, 221 to 450 as moderate activity, and >450 as severe activity) (28). Recurrence was defined as a CDAI of more than 220, an increase of at least 70 from the baseline between 150 and 220, or the need for a surgical procedure (29).

The quality of life of the patients was assessed using the Chinese translation of an Italian version of the Inflammatory Bowel Disease Questionnaire (IBDQ) (30), which is a widely recognized disease-specific questionnaire used to measure the quality of life. The IBDQ consists of 32 questions and four subscales, namely, bowel symptoms (e.g., bloody stools, abdominal pain), systemic symptoms (e.g., fatigue, sleep problems), emotional function (e.g., anxiety, anger, and depression), and social function (e.g., limited social activities, school, or work attendance). The score of each question ranges from 1 point (worst condition) to 7 points (best condition), with a total score ranging from 32 to 224, with a higher score indicating a better quality of life. All patients were asked to complete the IBDQ.

### Statistical analysis

Data were analyzed using the Statistical Package for the Social Sciences, version 26.0 for Mac (IBM SPSS Statistics 26). Continuous variables were presented as the mean ± standard deviation (SD), and the counting data were expressed as frequencies and percentages. The Mann–Whitney U-tests were used to compare the mean values between the two groups. The mean values of multiple groups were compared using a one-way analysis of variance. Factors affecting the quality of life were analyzed by multiple linear stepwise regression. The chi-square test and point-biserial correlation analysis were used to screen relapse-related factors, and binary logistic regression analysis was used to construct predictive models of relapse. To assess the performance of the resulting predictions, receiver operating characteristic (ROC) curves were plotted to calculate the area under the curve (AUC). A *P*-value of < 0.05 was considered statistically significant.

## Results

### Demographic and clinical characteristics of patients with IBD

A total of 212 questionnaires were distributed in the training cohort. Questionnaires were considered invalid if any question was unanswered. Finally, a total of 191 valid questionnaires were recovered, yielding a response rate of 90.1% (191/212). UC (132, 69.1%) was the most common type, followed by CD (55, 28.8%) and IBD-U (4, 2.1%). We only analyzed the data of patients with UC and CD to explore factors affecting the quality of life and recurrence. The demographic and clinical characteristics of patients are shown in Table 1. Among the patients with UC, 80 of them (60.6%) were men. The average age was 43.7 ± 12.8 years. The median duration of the disease was 6 years. EIMs occurred in 58 (43.9%) patients and 17 (12.9%) patients had complications. Among the patients with CD, 38 of them (69.1%) were men. The average age was 36.0 ± 13.5 years. The median duration of the disease was 7 years. A total of 24 (43.6%) patients had EIMs. Complications occurred in 24 (43.6%) patients.

**Table 1:** Demographic and clinical characteristics of patients with inflammatory bowel disease.

**Items**		**Training cohort**			**Verification cohort**		
		**IBD (*****n*** = **191)**	**UC (*****n*** = **132)**	**CD (*****n*** = **55)**	**IBD (*****n*** = **100)**	**UC (*****n*** = **61)**	**CD (*****n*** = **39)**
Sex, *n* (%)	Male	118 (61.8)	80 (60.6)	38 (69.1)	63 (63.0)	38 (62.3)	25 (64.1)
	Female	73 (38.2)	52 (39.4)	17 (30.9)	37 (37.0)	23 (37.7)	14 (35.9)
**Age**, ***n*** **(%)**							
	0–18	5 (2.6)	1 (0.8)	4 (7.3)	4 (4.0)	0 (0.0)	4 (10.3)
	18–35	66 (34.6)	39 (29.5)	26 (47.3)	41 (41.0)	20 (32.8)	21 (53.8)
	36–60	102 (53.4)	79 (59.8)	22 (40.0)	41 (41.0)	29 (47.5)	12 (30.8)
	>60	18 (9.4)	13 (9.8)	3 (5.5)	14 (14.0)	12 (19.7)	2 (5.1)
**Disease course (year)**, ***n*** **(%)**							
	< 5	85 (44.5)	62 (47.0)	20 (36.4)	58 (58.0)	37 (60.7)	21 (53.8)
	5–10	54 (28.3)	38 (28.8)	15 (27.3)	25 (25.0)	14 (23.0)	11 (28.2)
	>10	52 (27.2)	32 (24.2)	20 (36.4)	17 (17.0)	10 (16.4)	7 (17.9)
**Disease activity**, ***n*** **(%)**							
	Remission	17 (8.9)	7 (5.3)	9 (16.4)	5 (5.0)	0 (0.0)	5 (12.8)
	Mild	38 (19.9)	31 (23.5)	7 (12.7)	30 (30.0)	17 (27.9)	13 (33.3)
	Moderate	80 (41.9)	59 (44.7)	18 (32.7)	40 (40.0)	26 (42.6)	14 (35.9)
	Severe	56 (29.3)	35 (26.5)	21 (38.2)	25 (25.0)	18 (29.5)	7 (17.9)
Bowel resection, *n* (%)		30 (15.7)	1 (0.8)	28 (50.9)	33 (33.0)	10 (16.4)	23 (59.0)
Extraintestinal manifestations, *n* (%)		84 (44.0)	58 (43.9)	24 (43.6)	61 (61.0)	39 (63.9)	22 (56.4)
Complications, *n* (%)		41 (21.5)	17 (12.9)	24 (43.6)	57 (57.0)	34 (55.7)	23 (59.0)
Biological agents, *n* (%)		24 (12.6)	9 (6.8)	15 (27.3)	42 (42.0)	18 (29.5)	24 (61.5)
**Disease location**, ***n*** **(%)**							
(Montreal classification)	E1		29 (22.0)			14 (23.0)	
	E2		39 (29.5)			11 (18.0)	
	E3		64 (48.5)			36 (59.0)	
	L1			35 (63.6)			21 (53.8)
	L2			11 (20.0)			10 (25.6)
	L3			8 (14.5)			8 (20.5)
	L4			1 (1.8)			0 (0.0)
	B1			45 (81.8)			34 (87.2)
	B2			7 (12.7)			4 (10.3)
	B3			3 (5.5)			1 (2.6)
	Perianal lesions			10 (18.2)			12 (30.8)

IBD, inflammatory bowel disease; UC, ulcerative colitis; CD, Crohn's disease; and EIMS, extraintestinal manifestations.

### General characteristics of patients with IBD

In the training cohort (Table 2), 41 (31.1%) patients with UC were engaged in manual labor and 19 (14.4%) patients with UC felt that their occupation was stressful. The annual household income of 25 patients (18.9%) was < RMB¥ 10,000. Among the patients with CD, the occupation of 19 (34.5%) patients was manual labor, and 11 (20.0%) patients felt that their occupation was stressful. The annual household income of 11 patients (20.0%) with CD was < RMB¥ 10,000.

**Table 2:** General characteristics of patients with inflammatory bowel disease.

**Items**	**Training cohort**			**Verification cohort**		
	**IBD (*****n*** = **191)**	**UC (*****n*** = **132)**	**CD (*****n*** = **55)**	**IBD (*****n*** = **100)**	**UC (*****n*** = **61)**	**CD (*****n*** = **39)**
**Family size**, ***n*** **(%)**						
0–3	91 (47.6)	68 (51.5)	23 (41.8)	40 (40.0)	21 (34.4)	19 (48.7)
4–5	80 (41.9)	53 (40.2)	23 (41.8)	41 (41.0)	26 (42.6)	15 (38.5)
>5	20 (10.5)	11 (8.3)	9 (16.4)	19 (19.0)	14 (23.0)	5 (12.8)
**Marital status**, ***n*** **(%)**						
Unmarried	34 (17.8)	15 (11.4)	19 (34.5)	31 (31.0)	10 (16.4)	21 (53.8)
Married	146 (76.4)	108 (81.8)	35 (63.6)	66 (66.0)	48 (78.7)	18 (46.2)
Divorced	8 (4.2)	7 (5.3)	1 (1.8)	2 (2.0)	2 (3.3)	0 (0.0)
Death of a spouse	3 (1.6)	2 (1.5)	0 (0.0)	1 (1.0)	1 (1.6)	0 (0.0)
**Education background**, ***n*** **(%)**						
Primary school or below	27 (14.1)	12 (9.1)	14 (25.5)	13 (13.0)	13 (21.3)	0 (0.0)
Middle school and high school	65 (34.0)	50 (37.9)	13 (23.6)	38 (38.0)	23 (37.7)	15 (38.5)
College degree	92 (48.2)	67 (50.8)	24 (43.6)	43 (43.0)	22 (36.1)	21 (53.8)
Graduate degree	6 (3.1)	3 (2.3)	3 (5.5)	5 (5.0)	2 (3.3)	3 (7.7)
Uneducated	1 (0.5)	0 (0.0)	1 (1.8)	1 (1.0)	1 (1.6)	0 (0.0)
**Medical insurance**, ***n*** **(%)**						
Worker's health insurance	90 (47.1)	68 (51.5)	21 (38.2)	41 (41.0)	22 (36.1)	19 (48.7)
Medical insurance for urban residents	20 (10.5)	14 (10.6)	6 (10.9)	8 (8.0)	4 (6.6)	4 (10.3)
Commercial insurance	1 (0.5)	1 (0.8)	0 (0.0)	1 (1.0)	1 (1.6)	0 (0.0)
Student health insurance	4 (2.1)	2 (1.5)	2 (3.6)	2 (2.0)	1 (1.6)	1 (2.6)
Rural health care	72 (37.7)	43 (32.6)	26 (47.3)	48 (48.0)	33 (54.1)	15 (38.5)
Without health insurance	4 (2.1)	4 (3.0)	0 (0.0)	0 (0.0)	0 (0.0)	0 (0.0)
**Profession**, ***n*** **(%)**						
Manual labor	63 (33.0)	41 (31.1)	19 (34.5)	32 (32.0)	24 (39.3)	8 (20.5)
Mental work	66 (34.6)	49 (37.1)	16 (29.1)	31 (31.0)	15 (24.6)	16 (41.0)
Both manual labor and mental work	62 (32.5)	42 (31.8)	20 (36.4)	37 (37.0)	22 (36.1)	15 (38.5)
**Occupational stress**, ***n*** **(%)**						
Relaxed	39 (20.4)	25 (18.9)	11 (20.0)	19 (19.0)	12 (19.7)	7 (17.9)
Standard	122 (63.9)	88 (66.7)	33 (60.0)	58 (58.0)	35 (57.4)	23 (59.0)
Stressful	30 (15.7)	19 (14.4)	11 (20.0)	23 (23.0)	14 (23.0)	9 (23.1)
**Annual household income**, ***n*** **(%)**						
RMB¥ 10,000 or less	37 (19.4)	25 (18.9)	11 (20.0)	25 (25.0)	16 (26.2)	9 (23.1)
RMB¥ 10,000–50,000	67 (35.1)	46 (34.8)	21 (38.2)	36 (36.0)	25 (41.0)	11 (28.2)
>RMB¥ 50,000	87 (45.5)	61 (46.2)	23 (41.8)	39 (39.0)	20 (32.8)	19 (48.7)

IBD, inflammatory bowel disease; UC, ulcerative colitis; and CD, Crohn's disease.

### Quality of life scores in patients with IBD

We used the IBDQ to investigate the quality of life of patients with IBD (Table 3). The results showed that all dimensions of the IBDQ were decreased, with the total scores reduced by 23.2%. The score of systemic symptoms decreased the most. There were no significant differences in the score of intestinal symptoms, systemic symptoms, emotional functioning, and social functioning between UC and CD patients. It showed that the quality of life of patients with IBD was decreased but was not affected by the type of disease.

**Table 3:** Quality of life scores of patients with inflammatory bowel disease.

		**Scores (decline rate %)**			
**Dimensions**	**Total scores**	**IBD**	**UC**	**CD**	*P* ^a^
Bowel symptoms	10–70	55.8 ± 11.3 (20.2)	55.2 ± 11.1 (21.1)	56.7 ± 11.8 (18.6)	0.336
Systemic symptoms	5–35	24.9 ± 6.4 (28.9)	24.7 ± 6.2 (29.4)	25.0 ± 6.8 (28.6)	0.783
Emotional functioning	12–84	63.5 ± 12.5 (24.4)	63.3 ± 12.3 (24.7)	63.4 ± 13.2 (24.6)	0.967
Social functioning	5–35	27.9 ± 7.3 (20.3)	28.0 ± 7.2 (20.0)	27.4 ± 7.7 (21.6)	0.649
IBDQ total scores	32–224	172.2 ± 35.0 (23.2)	172.2 ± 34.4 (23.1)	172.8 ± 36.9 (22.9)	0.781

IBD, inflammatory bowel disease; UC, ulcerative colitis; CD, Crohn's disease; and IBDQ, Inflammatory Bowel Disease Questionnaire. ^*a*^The difference between ulcerative colitis and Crohn's disease; A *P*-value of < 0.05 was considered statistically significant.

### Univariate and multivariate analyses of factors affecting the quality of life of patients with UC and CD

This study analyzed the factors influencing the quality of life of patients with UC and CD (Table 4). The related factors with a *P*-value of < 0.1 in univariate analysis were included in the multiple regression analyses. Disease activity, age, EIMs, and annual household income were the factors that affected the total score of the IBDQ in patients with UC. Several factors affect intestinal symptoms, systemic symptoms, emotional function, and social function in patients with UC to varying degrees. Disease activity, EIMs, and occupational stress were the factors affecting the total scores and scores of all four dimensions of the IBDQ in patients with CD.

**Table 4:** Univariate analysis and multivariate stepwise regression analysis of factors affecting the quality of life of patients with ulcerative colitis and Crohn's disease.

	**Ulcerative colitis**					**Crohn's disease**				
**Dimensions**	**Univariate analysis** ^a^	**Multivariate analysis** ^b^				**Univariate analysis** ^a^	**Multivariate analysis** ^b^			
	***P***	***R** ^2^*	β	**t**	***P***	***P***	***R** ^2^*	β	**t**	***P***
IBDQ total scores		0.570					0.751			
Disease activity	< 0.001		−0.656	−10.978	< 0.001	< 0.001		−0.674	−9.119	< 0.001
Age	0.002		0.186	3.143	0.002	0.526				
Extraintestinal manifestations	0.022		0.135	2.228	0.024	0.001		0.253	3.440	0.001
Annual household income	0.064		0.124	2.118	0.036	0.016		0.067	0.904	0.370
Occupational stress	0.131					0.035		−0.251	−3.541	0.001
Profession	0.344					0.011		−0.101	−1.285	0.205
Education background	0.259					0.044		−0.045	−0.632	0.530
Complications	0.701					0.022		−0.029	−0.371	0.712
Bowel symptoms		0.473					0.607			
Disease activity	< 0.001		−0.628	−9.714	< 0.001	< 0.001		−0.588	−6.339	< 0.001
Age	0.001		0.203	3.149	0.002	0.360				
Extraintestinal manifestations	0.061		0.117	1.835	0.069	0.002		0.253	2.744	0.008
Occupational stress	0.335					0.064		−0.227	−2.540	0.014
Complications	0.721					0.046		−0.044	−0.451	0.654
Annual household income	0.150					0.008		0.108	1.175	0.246
Profession	0.252					0.022		−0.111	−1.115	0.270
Systemic symptoms		0.445					0.700			
Disease activity	< 0.001		−0.667	−10.202	< 0.001	< 0.001		−0.641	−7.912	< 0.001
Age	0.017		0.081	1.234	0.220	0.198				
Extraintestinal manifestations	0.043		0.117	1.790	0.076	0.001		0.259	3.213	0.002
Occupational stress	0.084		−0.097	−1.470	0.144	0.003		−0.244	−3.132	0.003
Complications	0.092		−0.109	−1.680	0.095	0.029		−0.018	−0.211	0.834
Profession	0.406					0.019		−0.046	−0.525	0.602
Annual household income	0.111					0.033		0.029	0.355	0.724
Education background	0.268					0.085		−0.038	−0.478	0.635
Emotional functioning		0.557					0.668			
Disease activity	< 0.001		−0.668	−11.134	< 0.001	< 0.001		−0.654	−7.673	< 0.001
Age	0.001		0.202	3.392	0.001	0.792				
Extraintestinal manifestations	0.033		0.118	1.983	0.050	0.005		0.213	2.509	0.015
Occupational stress	0.088		−0.090	−1.498	0.137	0.062		−0.229	−2.795	0.007
Complications	0.698					0.074		−0.017	−0.187	0.852
Profession	0.206					0.018		−0.157	−1.754	0.085
Annual household income	0.143					0.041		0.061	0.716	0.477
Education background	0.356					0.016		−0.093	−1.133	0.262
Social functioning		0.468					0.684			
Disease activity	< 0.001		−0.531	−7.948	< 0.001	< 0.001		−0.643	−7.721	< 0.001
Annual household income	0.034		0.177	2.706	0.008	0.064		0.024	0.282	0.779
Extraintestinal manifestations	0.018		0.162	2.467	0.015	0.002		0.231	2.794	0.007
Age	0.040		0.165	2.422	0.017	0.538				
Biological agents	0.050		0.206	3.062	0.003	0.601				
Occupational stress	0.212					0.043		−0.251	−3.140	0.003
Profession	0.648					0.004		−0.005	−0.060	0.952
Complications	0.870					0.013		−0.026	−0.298	0.767

^*a*^Univariate analysis was performed using Mann–Whitney U-tests and one-way analysis of variance. ^*b*^Multivariate analysis included variables with *P* < 0.1 in the univariate analysis for multiple stepwise regression analysis. A *P*-value of < 0.05 was considered statistically significant.

### Factors associated with the recurrence in patients with IBD

To analyze the factors associated with the recurrence of IBD in patients and construct a prediction model for disease recurrence, 191 patients with IBD in the training cohort were followed up for 1 year. In this period, these participants included 82 patients with recurrence, 99 patients without recurrence, and 10 (5.2%) patients lost to follow-up. The chi-square test and point-biserial correlation analysis were used to screen relapse-related factors. We included age, EIMs, profession, biological agents, complications, disease activity, annual household income, occupational stress, and IBDQ scores for analysis. Finally, several potential predictors for relapse were identified in our cohort. Disease activity, annual household income, occupational stress, and IBDQ scores were found to be associated with short-term recurrence in patients with IBD (Table 5).

**Table 5:** Factors associated with recurrence.

**Variables**	**X^2^/r**	***P***
Disease activity	18.195	< 0.001
Annual household income	21.708	< 0.001
Occupational stress	16.488	< 0.001
IBDQ total scores	−0.411	< 0.001
Age	4.052	0.256
Extraintestinal manifestations	2.383	0.123
Complications	3.910	0.059
Profession	4.693	0.096
Biological agents	1.023	0.312

IBDQ, Inflammatory Bowel Disease Questionnaire.

### The development of a model to predict recurrence

The relapse-related factors were included in the binary logistic regression analysis. The Hosmer–Lemeshow test was used to test the model fit and goodness, and the results showed that the model fit and goodness were good (*P* = 0.529). The linear predictor was 5.036 + (-0.026) × IBDQ + 1.976 × annual household income (RMB¥ 10,000 or less) + 0.898 × annual household income (RMB¥ 10,000 50,000) + (-2.067) × occupational stress (relaxed) + (-1.609) × occupational stress (standard) (Table 6). Predicted probabilities were calculated using the linear predictor in the formula: 1/(1+e^−*linearpredictor*^). When the *P*-value was >0.5, the patient was predicted to relapse. In this study, the model correctly predicted 72.9% of patient outcomes. The results revealed that 79.8% of the patients without recurrence predicted by the model did not have a recurrence and that 64.6% of the patients with recurrence predicted by the model had a recurrence.

**Table 6:** Associations between influencing factors and relapse of inflammatory bowel disease using binary logistic regression^a^.

**Variables**	**B value**	**SE**	***P***	**OR**	**95%cCI**
Occupational stress (Relaxed)^b^	−2.067	0.674	0.001	0.13	0.04–0.45
Occupational stress (Standard)^c^	−1.609	0.536	0.003	0.20	0.07–0.57
Annual household income (10,000 or less CNY)^d^	1.976	0.513	< 0.001	7.22	2.64–19.74
Annual household income (10,000–50,000 CNY)^e^	0.898	0.408	0.028	2.46	1.10–5.46
IBDQ total scores	−0.026	0.01	0.006	0.97	0.96–0.99
Constant	5.036	1.46	0.001	153.91	

B-value, regression coefficient; SE, standard error; OR, odds ratio; CI, confidence interval; IBDQ, Inflammatory Bowel Disease Questionnaire; ^*a*^Multivariable analysis included variables with a *P*-value of < 0.1 in the univariate analyses; ^*b*^occupational stress (relaxed) vs. occupational stress (stressful); ^*c*^occupational stress (standard) vs. occupational stress (stressful); ^*d*^annual household income (10,000 or less CNY) vs. annual household income (>50,000 CNY); and ^*e*^annual household income (10,000–50,000 CNY) vs. annual household income (>50,000 CNY).

### The ability to predict recurrence in patients with IBD

**Figure 2:**
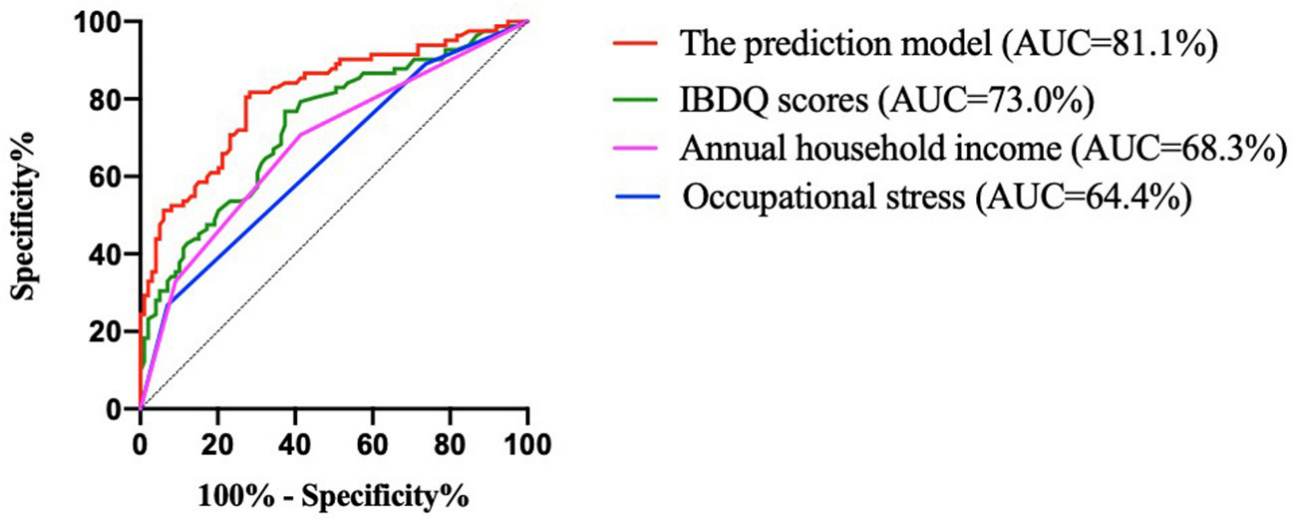
ROC curves for the developed model for predicting recurrence and variables. IBDQ, Inflammatory Bowel Disease Questionnaire; and AUC, area under the curve.

The discriminant ability was evaluated according to the AUC (Figure 2; Table 7). The results indicated that the predictive effect of the model was better than that of the independent predictors, and the predictive effect of the IBDQ scores on recurrence was better than that of annual household income and occupational stress. The AUC of the prediction model of this study was 81.1%, and the 95% confidence interval (CI) was 74.8–87.5%. The maximum value of the Youden index (a measure of the authenticity of a screening test, also known as the correctness index) was the best critical value of the prediction model. The Youden index of this study was 0.534. The sensitivity and specificity were 81.7 and 71.7%, respectively. The model could be considered to have a good discriminant effect.

**Table 7:** Predictive power of each variable and prediction model for recurrence.

**Variables**	**AUC**	***P***	**95% CI**	**Youden index**	**Sensitivity**	**Specificity**
Occupational stress	64.4%	0.001	0.563–0.724	0.197	0.268	0.929
Annual household income	68.3%	< 0.001	0.604–0.761	0.293	0.707	0.586
IBDQ scores	73.0%	< 0.001	0.656–0.804	0.394	0.768	0.626
Model	81.1%	< 0.001	0.748–0.875	0.534	0.817	0.717

AUC, area under the curve; CI, confidence interval; and IBDQ, Inflammatory Bowel Disease Questionnaire.

### Verification of the model

**Figure 3:**
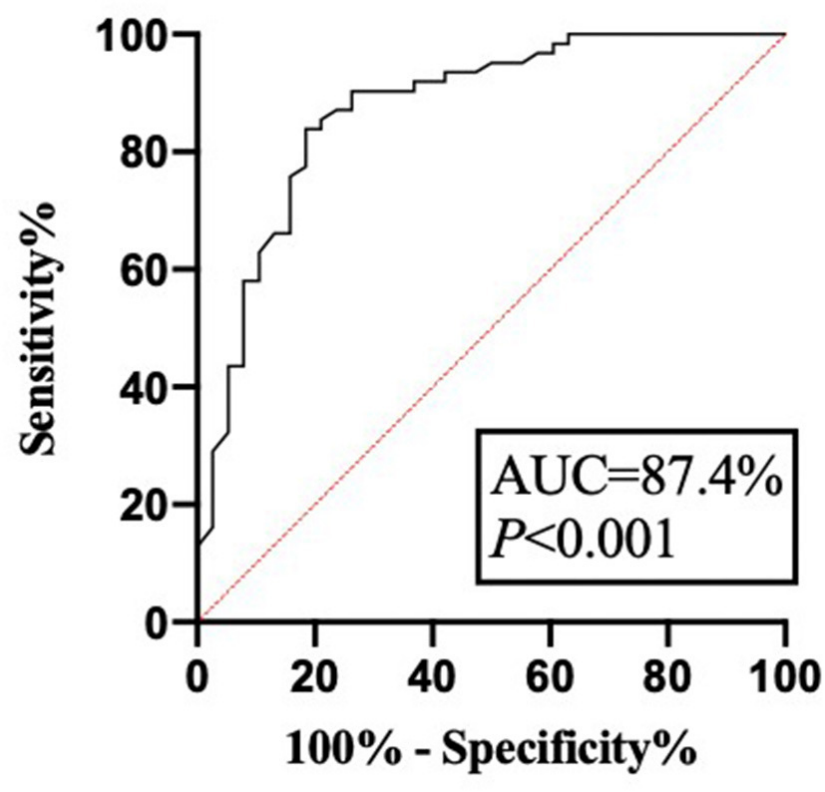
The ROC curve of model verification. AUC, area under the curve.

Another 100 patients with IBD diagnosed in the hospital from April 2021 to June 2021 were selected to establish a validation group. The demographic, clinical, and general characteristics of the patients in the validation cohort are shown in Tables 1, 2. Among these, 38 patients had recurrence within 1 year of follow-up and 62 patients had no recurrence. The successful modeling formula from the first stage was used to predict recurrence, with a sensitivity of 81.6%, a specificity of 83.9%, and an accuracy of 85.0%. The positive predictive value was 81.6%. The negative predictive value was 87.1%. The false-negative rate was 13.7%, and the false-positive rate was 20.5%. The ROC curve was drawn by the same method. The results revealed that the AUC was 87.4%, and the 95% CI was 79.9–94.8%. The Youden index was 0.655 (Figure 3). This indicated that the established recurrence prediction model also had good discriminant validity in the validation cohort.

## Discussion

Inflammatory bowel disease (IBD) is comprehensively affected by environmental factors, individual genotypes, intestinal microecology, autoimmunity, and other factors. Prognosis and recurrence prediction are of great significance for disease control. In recent years, the quality of life and social psychology of patients with IBD has drawn extensive concern. Due to the differences in clinical manifestations, treatment options, economic situation, cultural backgrounds, and lifestyles of patients with IBD, studies from other countries cannot reflect the quality of life and short-term recurrence of patients with IBD in China. In this study, patients with IBD in a tertiary hospital in Southwest China were selected as the research subjects. The results showed that the quality of life of patients with IBD was generally poor. Moreover, a prediction model of disease recurrence based on factors affecting the quality of life had a high predictive value. This study is of great significance for understanding the characteristics and quality of life of patients with IBD in China. The model established in this study can effectively guide treatment monitoring and follow-up.

The chronic progressive course, the side effects associated with medications, and the increasing financial burden reduce the quality of life of patients with IBD. A recent systematic review found that the quality of life of patients with IBD is worse than that of healthy people (31). We also found that patients with IBD had decreased quality of life in all dimensions, especially emotional functioning and systemic symptoms. This suggests that the therapeutic goals of IBD are not only mucosal healing but also an improved quality of life. Traditionally, patients with CD were believed to have a poorer quality of life than patients with UC due to worse disease behavior (32–34). Our study did not show a statistical difference between the two conditions. This may be because frequent diarrhea and bloody stool in patients with UC lead to lower intestinal symptom scores, and these symptoms are more likely to affect the physical health and social activities of the patients. In addition, patients with CD had a higher proportion of biologics than patients with UC in our study. Although our data revealed no significant association between biologics and the quality of life, Zhang et al. (35) found that the use of biologics had a positive effect on the quality of life of patients with CD. The differences may be due to the heterogeneity of patients and studies. Most patients treated with biologics have severe or complex conditions, and their emotional experiences may be completely different in the early stages of treatment and the period after disease control. This suggests that both UC and CD seriously affect the quality of life. Multivariate analysis showed that the independent factors influencing the quality of life of patients with UC were disease activity, annual household income, EIMs, and age, which explained ~57.0% of the quality of life score. However, disease activity, occupational stress, and EIMs were independent factors influencing the quality of life of patients with CD, accounting for ~75.1%.

In our study, disease activity affecting all dimensions of the IBDQ was an independent predictor of poor IBDQ scores, and the amount of explanation reached 50.7% (UC) and 62.3% (CD), respectively. This finding was consistent with other studies (36, 37). Zhao et al. (38) found that high disease activity scores were associated with the early recurrence of IBD after fecal bacteria transplantation. Our data also confirmed that disease activity was associated with the recurrence of IBD. Patients in remission or relapse experience different levels of anxiety, depression, sleep problems, and stress (39). Effective treatment and psychological intervention can reduce disability and improve the quality of life of patients with IBD (40). The reported prevalence of EIMs ranges from 6 to 47% (41). These EIMs regularly result in significant morbidity in patients with IBD, even more so than the intestinal disease itself (42). We found that EIMs simultaneously affected the four dimensions of the quality of life of patients with CD and affected the total IBDQ scores by reducing the social functioning scores in patients with UC. Ott et al. (43) also found that EIMs significantly influence the quality of life. Multidisciplinary management of IBD and EIMs can simultaneously improve outcomes and the quality of life (44). Therefore, disease activity and EIMs should be assessed in patients with IBD on a regular basis as prevention and/or specific treatment can have a major benefit on patients' quality of life and outcomes. Although it was recognized that elderly patients have poorer physiological conditions and higher treatment risks, univariate analysis reported that age could affect several dimensions of the IBDQ in patients with UC, and it was confirmed that age could affect the total IBDQ score in multivariate analysis. Advanced age did positively impact the quality of life of patients with UC, but it was not evident in CD. Perera et al. also confirmed that advanced age did not have a negative impact on the health-related quality of life of patients with IBD (45). The elderly population may receive more social and family support to cope with the condition. We found that low annual household income reduced total IBDQ scores in patients with UC. Two studies, by Liu et al. (46) and Yoon et al. (47), found that annual income was an independent predictor of reduced quality of life of patients with IBD. In addition, low annual household income was found to be a risk factor for disease recurrence in our predictive model. This may be because patients with low income experienced more stress and had fewer treatment options available. Patients with IBD are often impaired in their ability to be employed due to their morbidity. A study from Germany showed that more than half of the patients with IBD had a negative subjective prognosis for employment and experienced daily work-related problems, including reduced work ability, fear of not being fully productive, and work stress (48). Occupational stress was associated with the total IBDQ scores reflecting a decrease in all dimensions in patients with CD based on our results. Occupational stress was an independent risk factor for disease recurrence. Compared to patients with high occupational stress, patients with the standard [OR 0.20 (0.07–0.57)] and relaxed [OR 0.13 (0.04–0.45)] occupational stress had a lower risk of recurrence. For this complex condition, rehabilitation programs and support services that meet patients' needs should be implemented for work-related problems.

At least ~10–50% of patients with CD undergo one or more surgical procedures during their lifetime and ~5–10% of patients with UC require surgery within 5 years (10, 49). Studies suggest that surgery and the use of biologics were associated with the quality of life of patients with IBD (47, 50). Another study found that lower education levels and socioeconomic levels were related factors for the poor quality of life of patients with IBD (51). Our multivariate analysis did not show that intestinal surgery or biologics were independent predictors of the quality of life. This can be explained by the low rate of surgery and the use of biologics. In univariate analysis, we found that complications, profession, and educational background could affect several dimensions of scores of the IBDQ, but there was no statistical significance in multivariate analysis. Several studies have reported a significant correlation between the quality of life and the duration of disease, indicating better quality of life with a greater duration of IBD (50, 52, 53). Knowles et al. argued that, although symptoms persisted, patients no longer reacted negatively to them, viewing this condition as the new normal (54). This change reflects a process of self-adaptation to the chronic condition (55). Our results did not indicate a relationship between the duration of diseases and the quality of life. This may be because the time of our study was concentrated, and long-term longitudinal changes in patients were not visible.

Previous studies rarely considered the impact of disease burden on the recurrence of IBD. In this study, binary logistic regression analysis indicated that the quality of life, occupational stress, and annual household income were independent risk factors for recurrence, among which the quality of life was the most significant factor affecting recurrence. The higher the IBDQ scores, the lower the recurrence probability. Previous studies suggested that relapse-prone patients had a lower quality of life than patients in long-term remission (56, 57). This study found that patients with poor quality of life were more likely to relapse in the short term. These psychological factors will not trigger IBD, but they may have a negative effect on the progression and recurrence of the disease (58, 59). Patients with IBD with lower quality of life and higher occupational stress may be more likely to experience more anxiety, depression, sleep problems, and stress, and thus be more likely to relapse. Studies revealed that the medication compliance of patients with IBD is generally poor, ranging from 25.0 to 40.9%. Poor treatment compliance was closely related to recurrence (60, 61). Low annual household income may be a key factor affecting the treatment compliance of patients, resulting in a relatively high recurrence rate in the short term. The prediction model established by the risk factors affecting the quality of life had a good discriminative effect and could effectively screen for a population at high risk of recurrence. Our study analyzed the four dimensions of the IBDQ and found that they were affected by different factors. Compared with CRP, FC, ESR, endoscopy, or pathology scores, the model established based on the factors influencing the quality of life in this study is more practical, non-invasive, and cost-effective. Clinicians should pay more attention to the quality of life, occupational stress, and low income of patients with IBD. Early detection of patients at high risk of recurrence, as well as enhanced education and follow-up, will have a positive effect on disease control, show an improvement in the quality of life, and lead to a reduction of recurrence in patients with IBD.

This study has several limitations. The long-term quality of life and recurrence rate were not available during the observation period of this study. Due to the differences in socioeconomic factors, the cutoff values of factors affecting the quality of life, such as income, need to be adjusted. In addition, questionnaire participants in this study were mainly young and middle-aged, with the elderly and children being underrepresented. To reduce the selection bias, the study randomly investigated hospitalized patients with IBD at an IBD center and a tertiary hospital in Southwest China. Therefore, the results of the study are still reliable.

In conclusion, quality of life and recurrence rate are prognostic indicators of patients with IBD. The prediction model of recurrence based on factors affecting the quality of life can support the management of IBD by selecting patients at a higher risk of relapse. Interventions targeting the four dimensions of quality of life can be beneficial in reducing relapse and improving the quality of life of patients with IBD. Subsequently, longitudinal studies on the quality of life throughout treatment and after interventions can provide effective social support approaches and optimal management strategies for patients with IBD.

## Data availability statement

The original contributions presented in the study are included in the article/supplementary material, further inquiries can be directed to the corresponding authors.

## Ethics statement

The study involving human participants was approved by the Ethics Committee of Kunming Medical University, Kunming, Yunnan province, China (the approval number: KMMU20192032). Informed consent was obtained from all the included patients.

## Author contributions

JN, YM, ML, and YT contributed to the study design. ML, YT, YS, JW, FZ, YW, MG, JY, HL, and XB wrote and revised the manuscript. JN and YM reviewed the manuscript. All the authors have read and approved the final version of the manuscript.
